# Supplementary material for: HIV-1 vaccination by needle-free oral injection induces strong mucosal immunity and protects against SHIV challenge
Source: Nat Commun. 2019 Feb 18;10:798. doi: 10.1038/s41467-019-08739-4 (PMC6379385; doi:10.1038/s41467-019-08739-4)
Supplement: Supplementary file 1 — Supplementary Information [file 41467_2019_8739_MOESM1_ESM.pdf]

Jones et al. **HIV-1 vaccination by needle-free oral injection induces strong mucosal immunity and protects against SHIV challenge**

Supplementary Information

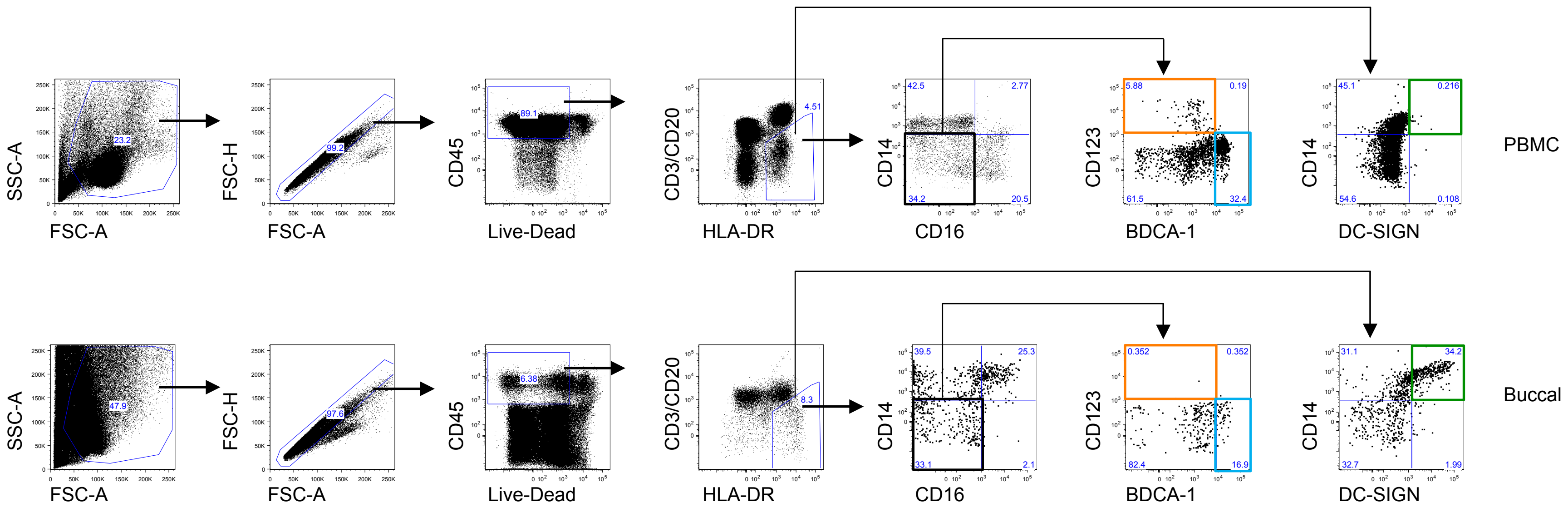

Supplementary Figure 1: Gating strategy for dendritic cells in PBMCs and tissues. Representative gating for conventional dendritic cells (CD45$^+$ CD3$^-$ CD20$^-$ HLA-DR$^+$ CD14$^-$ CD16$^-$ BDCA-1$^+$ CD123$^-$ Live cells), plasmacytoid dendritic cells (CD45$^+$ CD3$^-$ CD20$^-$ HLA-DR$^+$ CD14$^-$ CD16$^-$ BDCA-1$^-$ CD123$^+$ Live cells), and dermal dendritic cells (CD45$^+$ CD3$^-$ CD20$^-$ HLA-DR$^+$ CD14$^+$ DC-SIGN$^+$ Live cells) in PBMCs (top) and buccal tissue (bottom). Sublingual tissue, submandibular lymph nodes, submental lymph nodes, and inguinal lymph nodes are gated similarly. Blue gate, conventional DCs; orange gate, plasmacytoid DCs; green gate, dermal DCs.

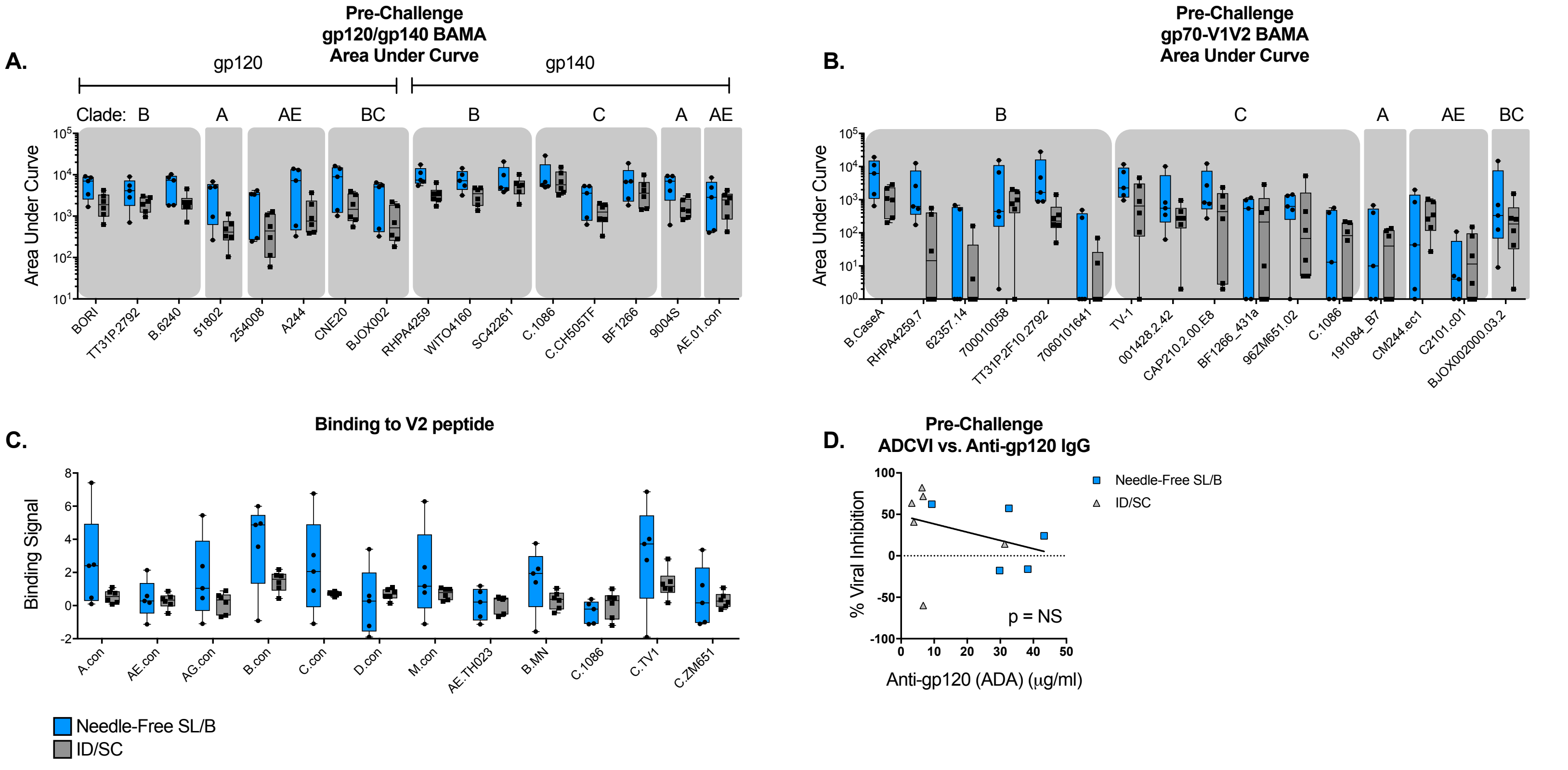

Supplementary Figure 2: Antibody magnitude, specificity, and effector functionality. BAMA analysis of ID/SC and needle-free SL/B immunized animal serum IgG at time of pre-challenge (wk 45) against gp120/gp140 antigens (**A**) and gp70-V1V2 antigens (**B**), quantified as Area Under Curve (AUC) analysis. HIV-1 Env strains organized by clade (B, C, A, AE, BC). (**C**). Serum IgG (wk 25) binding to linear V2 peptides from consensus clade A, AE, AG, B, C, D, group M, and viral strains AE.TH023, B.MN, 1086.C, C.TV-1, and C.ZM651, measured by peptide microarray linear epitope mapping and reported as Binding Signal (Log2 fold difference post-immunization/baseline binding intensity). (**D**) Correlation analysis comparing ADCVI activity to anti-gp120 IgG in serum at pre-challenge in needle-free SL/B (blue square) and ID/SC (grey triangle) immunized animals. Spearman correlation test. (**A-C**) Box and whiskers plot; box extends from 25th to 75th percentile, line indicates median, whiskers indicate min and max values. Blue shade, needle-free SL/B (n=5), grey shade, ID/SC (n=6).

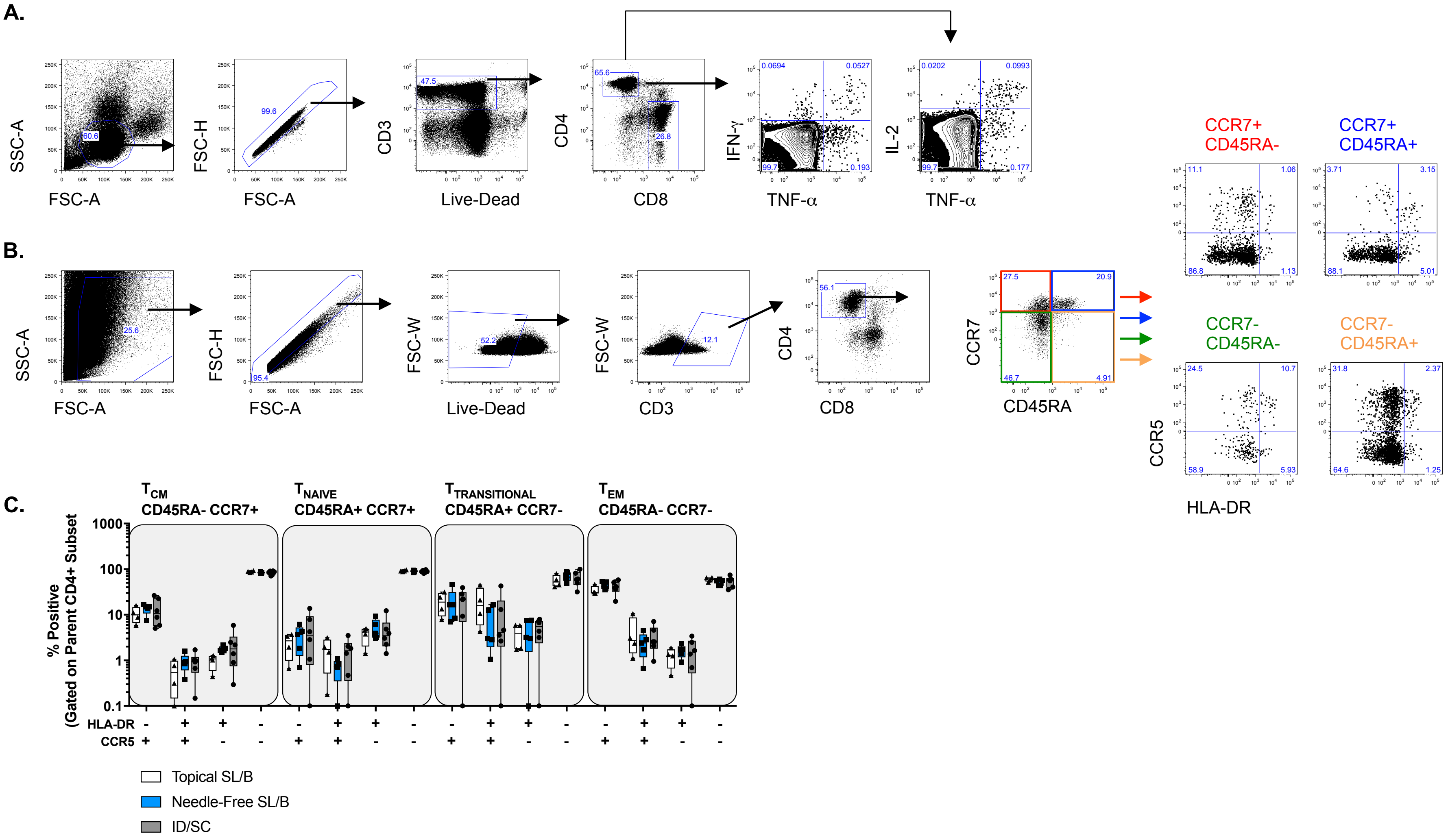

Supplementary Figure 3: T-cell gating and phenotype. (A) Gating strategy for CD4⁺ and CD8⁺ T-cells (Live CD3⁺) cells in PBMCs, gated for IFN-γ, TNF-α, and IL-2 expression. Cytokine plots represent Env-peptide stimulated CD4⁺ T-cells from needle-free SL/B immunized animal (wk 25). (**B**) Gating strategy for rectal CD4⁺ T-cell subsets (Live CD3⁺CD4⁺). Central memory (T_{CM}) ($CCR7^+CD45RA^-$) (Red), Naïve ($CCR7^+CD45RA^+$) (Blue), Transitional memory ($CCR7^-CD45RA^+$) (Orange) and effector memory (T_{EM}) or effector ($CCR7^-CD45RA^-$) (Green) CD4⁺ subsets were gated for CCR5 and HLA-DR expression. (**C**) Frequencies of HLA-DR and CCR5 expressing CD4+ T-cell subsets in rectum, taken at the pre-challenge time point (wk 45). Box and whiskers plot; box extends from 25th to 75th percentile, line indicates median, whiskers indicate min and max values. White shade, Topical SL/B (n = 4), Blue shade, needle-free SL/B (n=5), grey shade, ID/SC (n=6).

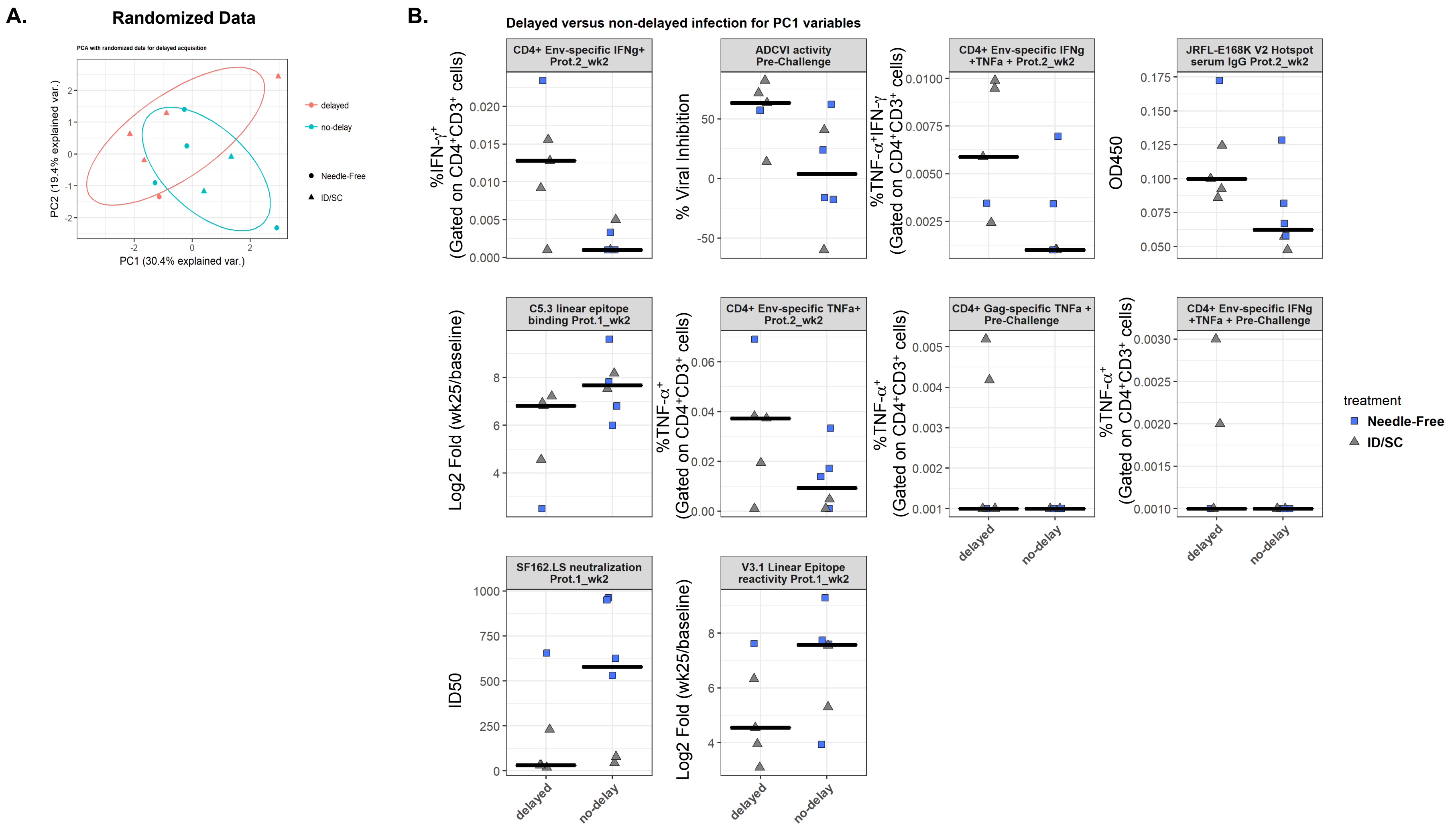

Supplementary Figure 4: Principal component analysis of immune parameters in delayed and non-delayed infected animals. (A) PCA performed as in Fig. 6d with randomized assay data. Animals separated into delayed (≥ 5 challenges to be infected) (n=5) vs. non-delayed (< 5 challenges to be infected) (n=6) groups. (**B**) Comparison of immune parameters included in the principal component 1 (PC1) of the PCA, separated by animals showing delayed or non-delayed SHIV-SF162P3 acquisition. Line indicates median. Blue square, needle-free SL/B; grey triangle, ID/SC

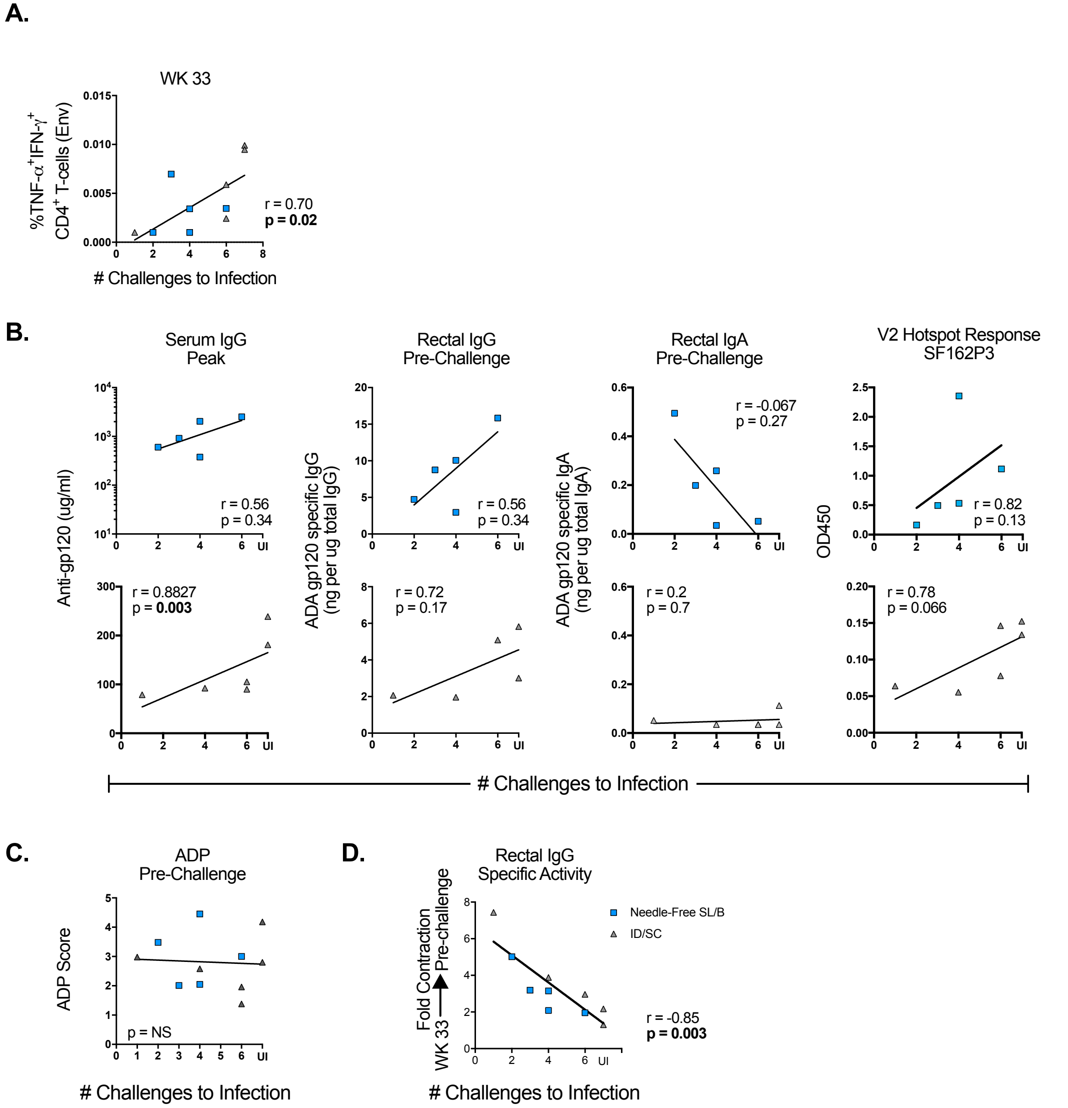

Supplementary Figure 5: Correlates of protection against SHIV-SF162P3 infection. (A) Correlation analysis of %TNF-α⁺IFN-γ⁺ CD4⁺ T-cells in response to Env peptide stimulation two weeks post the second protein boost (wk 33) and acquisition of infection. Both groups, needle-free SL/B and ID/SC are combined (n=11). (**B**) Correlation analysis of peak serum (wk 25) gp120 specific serum IgG, gp120 specific activity of rectal IgG and IgA at the pre-challenge timepoint (wk 45), and peak SHIV-SF162P3 V2 hotspot responses (WK 25, Needle-free oral; WK 33, ID/SC) and acquisition of infection. Vaccine groups separated by needle-free SL/B (blue square, n=5) and ID/SC (grey triangle, n = 6). (**C**) Correlation analysis of ADP activity at pre-challenge and acquisition of infection. Groups combined. (**D**). Correlation analysis comparing the fold contraction of gp120-specific rectal IgG (specific activity) from two weeks post the second protein boost (wk 33) to the pre-challenge time point (wk 45) and acquisition of infection. Groups combined. (**A-D**) Spearman correlation analysis.

Epitope	Peptide Region	AA Range (HXB2 Nbr)
C1.1	#34-35	aa101-118
V2	#54-55	aa166-183
C2.1	#66-67	aa202-219
C2.2	#82-84	aa250-270
C2.3	#88-89	aa268-285
V3.1	#99-101	aa301-321
V3.2	#102-103	aa312-329
C3	#122-123	aa373-390
C4	#136-138	aa424-444
V5	#148-149	aa460-477
C5.1	#152-153	aa472-489
C5.2	#157-159	aa487-507
C5.3	#160-161	aa496-513

Supplementary Table 1: Linear epitope mapping peptide positions in HIV-1 reference strain HXB2.
