# Supplementary material for: HIV-1 vaccination by needle-free oral injection induces strong mucosal immunity and protects against SHIV challenge
Source: Nat Commun. 2019 Feb 18;10:798. doi: 10.1038/s41467-019-08739-4 (PMC6379385; doi:10.1038/s41467-019-08739-4)
Supplement: Supplementary file 3 — Reporting Summary [file 41467_2019_8739_MOESM3_ESM.pdf]

nature research

Corresponding author(s): Rama Rao Amara

Reporting Summary

Nature Research wishes to improve the reproducibility of the work that we publish. This form provides structure for consistency and transparency in reporting. For further information on Nature Research policies, see Authors & Referees and the Editorial Policy Checklist.

Statistical parameters

When statistical analyses are reported, confirm that the following items are present in the relevant location (e.g. figure legend, table legend, main text, or Methods section).

n/a	Confirmed	
☐	☒	The exact sample size (n) for each experimental group/condition, given as a discrete number and unit of measurement
☐	☒	An indication of whether measurements were taken from distinct samples or whether the same sample was measured repeatedly
☐	☒	The statistical test(s) used AND whether they are one- or two-sided<br>*Only common tests should be described solely by name; describe more complex techniques in the Methods section.*
☐	☒	A description of all covariates tested
☐	☒	A description of any assumptions or corrections, such as tests of normality and adjustment for multiple comparisons
☐	☒	A full description of the statistics including central tendency (e.g. means) or other basic estimates (e.g. regression coefficient) AND variation (e.g. standard deviation) or associated estimates of uncertainty (e.g. confidence intervals)
☐	☒	For null hypothesis testing, the test statistic (e.g. F, t, r) with confidence intervals, effect sizes, degrees of freedom and P value noted<br>*Give P values as exact values whenever suitable.*
☒	☐	For Bayesian analysis, information on the choice of priors and Markov chain Monte Carlo settings
☐	☒	For hierarchical and complex designs, identification of the appropriate level for tests and full reporting of outcomes
☒	☐	Estimates of effect sizes (e.g. Cohen's d, Pearson's r), indicating how they were calculated
☐	☒	Clearly defined error bars<br>*State explicitly what error bars represent (e.g. SD, SE, CI)*

Our web collection on statistics for biologists may be useful.

Software and code

Policy information about availability of computer code

Data collection	BD FACSDIVA was used to collect flow cytometry data and FlowJo 9 was used to analyze flow cytometry data.
Data analysis	GraphPad Prism was used to graph data and to perform statistical tests. Additional univariate correlation analysis was done using R and confirmed using SAS. Principal component analysis (PCA) was performed in R using the precomp package.

For manuscripts utilizing custom algorithms or software that are central to the research but not yet described in published literature, software must be made available to editors/reviewers upon request. We strongly encourage code deposition in a community repository (e.g. GitHub). See the Nature Research guidelines for submitting code & software for further information.

Data

Policy information about availability of data

All manuscripts must include a data availability statement. This statement should provide the following information, where applicable:

- Accession codes, unique identifiers, or web links for publicly available datasets
- A list of figures that have associated raw data
- A description of any restrictions on data availability

All data can be made available upon request

Field-specific reporting

Please select the best fit for your research. If you are not sure, read the appropriate sections before making your selection.

☒ Life sciences ☐ Behavioural & social sciences ☐ Ecological, evolutionary & environmental sciences

For a reference copy of the document with all sections, see nature.com/authors/policies/ReportingSummary-flat.pdf

Life sciences study design

All studies must disclose on these points even when the disclosure is negative.

Sample size	Sample sizes were determined based on historical non-human primate vaccine studies
Data exclusions	No data was excluded
Replication	All antibody binding assays were performed in duplicates. Flow cytometry analysis of rhesus macaque dendritic cell subsets was derived from several individual animals. Functional antibody assays such as ADCVI were performed twice and the average of two experiments is depicted.
Randomization	Animals were randomized across experimental groups
Blinding	The veterinary staff performing immunizations and sampling animals were blinded to the vaccine study design. Mucosal antibody binding assays, BAMA, and neutralizing antibody assays were performed under blinded conditions initially.

Reporting for specific materials, systems and methods

Materials & experimental systems

n/a	Involved in the study
☒	☐ Unique biological materials
☒	☐ Antibodies
☒	☐ Eukaryotic cell lines
☒	☐ Palaeontology
☒	☐ Animals and other organisms
☒	☐ Human research participants

Methods

n/a	Involved in the study
☒	☐ ChIP-seq
☐	☒ Flow cytometry
☒	☐ MRI-based neuroimaging

Flow Cytometry

Plots

Confirm that:

☒ The axis labels state the marker and fluorochrome used (e.g. CD4-FITC).

☒ The axis scales are clearly visible. Include numbers along axes only for bottom left plot of group (a 'group' is an analysis of identical markers).

☐ All plots are contour plots with outliers or pseudocolor plots.

☒ A numerical value for number of cells or percentage (with statistics) is provided.

Methodology

Sample preparation	Cells were isolated from rhesus macaque whole blood or tissues as described in the methods section.
Instrument	All flow cytometry assays were acquired using a BD LSRFortessa
Software	BD FACSDIVA was used to acquire data
Cell population abundance	We did not perform cell-sorting in these experiments
Gating strategy	An initial gate was placed on a SSC-A vs. FSC-A plot. From this singlets were gated using FSC-H vs. FSC-A. Singlets were then gated for live CD45+ cells in a CD45 vs. Live-Dead plot. Live CD45+ cells were then gated for CD3-CD20-HLA-DR+ in a CD3/CD20 vs. HLA-DR plot. Conventional DCs (cDCs) were then gated for CD14-CD16- in a CD14 vs CD16 plot, followed by BDCA1 vs CD123,

with BDCA-1 cells denoting cDCs. Dermal DCs were gated on CD3-CD20-HLA-DR+ cells followed by CD14 vs DC-SIGN, with CD14 +DC-SIGN+ cells denoting dermal DCs.

☒ Tick this box to confirm that a figure exemplifying the gating strategy is provided in the Supplementary Information.
